# Understanding the consumers’ multi-competing brand community engagement: A mix method approach

**DOI:** 10.3389/fpsyg.2022.1088619

**Published:** 2023-02-08

**Authors:** Kai He, Junyun Liao, Fengyan Li, Hongguang Sun

**Affiliations:** ^1^Jinan University, Guangzhou, China; ^2^Research Institute on Brand Innovation and Development of Guangzhou, School of Management, Jinan University, Guangzhou, China; ^3^School of Business, Macau University of Science and Technology, Macau, China

**Keywords:** brand community, competing brand communities, consumer motivation, consumer knowledge, brand community-swinging, netnography

## Abstract

**Introduction:**

Participating in multiple competing brand communities simultaneously is common for consumers, which brings challenges for companies to manage brand communities and build strong brand-consumer relationships. Although previous studies have widely examined the drivers and outcomes of consumers’ engagement in an individual community, little is known about the multi-competing brand community engagement.

**Methods:**

This paper explores the manifestation, categories, motivational drivers, and consequences of consumers’ MBCE through two studies using two different methodologies to fill this gap.

**Results:**

By using netnography, study 1 shows that MBCE behaviors manifest in various ways, and can be classified into three categories: information-oriented MBCE, social-oriented MBCE, and oppositional MBCE. Study 2 indicates through a consumer survey that one reason that motivates consumers to participate in other competing brand communities is because of the attractiveness of other competing brands. Also, the results indicate that consumers’ product knowledge is positively associated with MBCE. Finally, the number of competing brand community engagements is positively related to brand switching intention.

**Discussion:**

This article enriches the brand community literature and provides important implications on managing brand communities in a competing environment.

## Introduction

Realizing the importance of brand communities, an increasing number of brands have established brand communities to interact and communicate with consumers (Chen and Liao, 2022; Hsieh et al., 2022). Brand communities have also become a critical channel for consumers to obtain information and build relationships with each other through social interactions (Wu et al., 2018). With the rapid development of the internet, many firms have built their brand communities online, which then became the so-called virtual brand communities (Wu et al., 2015; Akrout and Nagy, 2018; Liao et al., 2020). As an indispensable part of the internet, social media platforms allow brands to establish their own brand communities and interact with their community members conveniently, thus attracting marketers to use these social-platform based brand communities to improve relationships with consumers (Demiray and Burnaz, 2019; Jeong et al., 2021). Such social media platform-based brand communities can directly utilize the network infrastructure of social media platforms, which is low in construction and maintenance costs and is easy to manage and operate (Palazon et al., 2018; Demiray and Burnaz, 2019). Thus, more firms have built their virtual brand communities on social media platforms (Sanz-Blas et al., 2019).

The existence of various brand communities on social media platforms allows users to be active on multiple competing brand communities, which makes firms face the challenge of retaining customers and understand the brand-switching behaviors of customers (Garga et al., 2019; Tandoc et al., 2019). For example, many competing brands in the same industries (e.g., smartphone brands such as Xiaomi and Huawei) have established their brand communities in a famous social media platform in China called Baidu Tieba. The openness of third-party social media platforms enables users to participate in various brand communities at will to obtain product information or engage in social interactions and even switch between different brand communities (Tandoc et al., 2019). An increasing number of scholars have found that instead of participating in a single brand community, a large number of consumers tend to participate in and navigate through different brand communities belonging to multiple brands within an industry, even the competing brands (Thompson and Sinha, 2008; Tan and Lee, 2015; Thompson et al., 2016; Liao et al., 2022). This phenomenon is defined as “multi-competing brand community engagement” (MBCE). Formally, MBCE refers to consumers’ engagement in several virtual brand communities belonging to two or more competing brands in an industry (e.g., OPPO and Xiaomi in the smartphone industry) within a social media platform during a certain period.

Previous studies of brand communities mostly focused on consumers’ engagement in a single brand community (Hollebeek et al., 2019; Kumar and Kumar, 2020). For instance, Kaur et al. (2020) revealed the effect of the consumers’ brand community identification on their engagement in the focal brand community. However, the consumers’ engagement and mobility among multi-competing brand communities pose serious challenges for companies to interact with consumers and build stable brand-consumer relationships, urging companies to take competing brand communities into consideration when managing brand communities (Miller et al., 2009; Ransbotham and Kane, 2011; Liao et al., 2021b). Because MBCE may bring various consequences to brand communities, including the reduction of the focal brand community’s user base and attraction of new users for other brand communities, it has become an issue of great interest to academics (Wang et al., 2012; Thompson et al., 2019). Tan and Lee (2015) stated that multi-community engagement is an underexploited research area and related studies can provide important implications for brand community management. Kumar and Nayak (2019) indicated that scholars should conduct studies to explore consumers’ psychological motivations for their engagement in multiple brand communities. As such, this paper aims to uncover the types of MBCE and consumers’ motivations for MBCE by conducting two studies. We explore and classify the behavioral characteristics of MBCE through netnography (Study 1) and explore the motivations and outcomes of MBCE by conducting a consumer survey (Study 2).

This study provides several important theoretical contributions. First, this article indicates that a brand community does not exist individually, but in a competing environment with other competing brand communities, enriching the literature on consumers’ brand community engagement. By distinguishing the types and motivations outcomes of consumers’ participation in multiple brand communities rather than a single brand community, this study provides a comprehensive understanding of consumers’ community engagement behavior in academia (Liao et al., 2022). This study offers a new perspective on brand community research by considering the role of competing brand communities (Tandoc et al., 2019). Thus, several important practical implications for companies to manage brand communities in terms of customer inflow and churn are also discussed. Overall, this research provides new insights into the antecedents and outcomes of MBCE.

## Literature review

To connect with customers, multiple brands have established online communities to develop stronger relationships with consumers (Baldus et al., 2015). Participating in different brand communities at the same time is common for consumers due to the coexistence of multiple brand communities (Tandoc et al., 2019). However, research in MBCE has been remarkably sparse to date. Thompson and Sinha (2008) explored the impact of membership overlap (participation in multiple brand communities), which demonstrates that engagement in the focal brand community fostered consumers to adopt the rival products when the focal brand lacked a counterpart compared wtih the competitor’s product. This study provides initial insight into the impact of multiple brand community engagement. However, they ignore the differential manifestation, types, and drivers of MBCE. Thompson et al. (2016) reported the effect of competing brand community engagement on consumers’ helping behavior toward other members. They documented that higher levels of engagement in the focal brand community may increase the likelihood of adopting products from rival brands. Wang et al. (2012) investigated the impact of membership overlap among online communities on their growth scale based on organizational ecology. The study offers valuable insights that sharing membership with other online communities would reduce the growth of focal online community scale and this negative effect is more pronounced in large online communities. Interestingly, Zhu et al. (2014) have shown that membership overlap has a positive effect on the survival of online communities. Compared with new online communities, sharing members with other mature online communities are more likely to develop the focal brand community. Kim et al. (2018) demonstrated that, membership overlap in online communities generally brings about competition for time allocation. However, under the presence of both external bridging and internal bonding, MBCE can lead to the improvement of community responsiveness. These studies focus on general interest communities such as Usenet newsgroups (Wang et al., 2012) or knowledge communities such as Wiki communities (Zhu et al., 2014) which do not have direct competition between brand communities and potential conflicts in the identity consciousness of their members (Thompson and Sinha, 2008; Kozinets et al., 2010; Thompson et al., 2016).

The aforementioned studies initially explore consumer mobility in multiple brand communities and its impact, but it is noteworthy that they do not answer why consumers participate in other competing communities and what types of these MBCEs are included. Baldus et al. (2015) indicated that there has been little research on the motivations of consumers to engage in these communities continually. Liao et al. (2022) stated that information value and social interaction value are strong antecedents of brand community-swinging. Consumers would be inclined to participate in multiple competing brand communities because of purchase decisions and information value but they leave because they have obtained sufficient information. Brand communities are not only an important channel for consumers to reduce information search costs and perceived risk, but also a group gathering place containing consumers’ interactive experience and value co-creation in the age of social media (Brodie et al., 2013; Liao et al., 2017). Zaglia (2013) assumed that the antecedents of participation in the brand communities include willingness to improve skills, social relation to others, and social position enhancement. Oppositional brand loyalty in brand communities motivates member loyalty to the preferred brand and resistance to the competing brands (Kuo and Feng, 2013). Community members promote the preferred brand, but they deride and mock the competitor brands out of oppositional brand loyalty. Engagement in groups with strong negative feelings toward a competing brand allows members to fuel feelings of belonging (Osuna Ramírez et al., 2019). These studies provided us with some directions to explore the categories and motivations of MBCE.

If consumers have varying motivations to participate in multi-competing brand communities, their engagement will probably generate different influences. Analyzing consumers’ motivations to participate in multi-competing brand communities helps toward understanding the reasons for consumers’ switching and churning, and provides firms with clear guidance on brand community member management.

## Study 1: Exploring the manifestation and categories of multi-competing brand community engagement

### Netnography

This study aims to reveal the manifestation and categories of MBCE using the netnography approach. Because of the absence of a conceptualization and the lack of understanding of the nature of MBCE in the academic community, it is appropriate to use qualitative research as our research method to explore its nature. In online contexts, Kozinets (2002) developed netnography for online social interaction research based on ethnography in sociology. Netnography is faster, simpler, and more economical than ethnography and focuses on group interviews, which directly use real data generated by online consumer interactions (Kozinets, 2002). This research method is extremely advantageous in revealing the symbolic interactions, and consumption patterns of online consumer groups. Netnography has been widely used in online community research related to brand rivalry and community conflict (Muñiz and Schau, 2007; Ewing et al., 2013).

### Data collection and analysis

This research focuses on the smartphone industry for the following reasons. First, there are many smartphone brands and the competition is fierce within the industry (Liao et al., 2021a). Most brands establish their brand communities, which provides the possibility to observe consumers’ MBCE to attract users. Because smartphones are highly technical products, consumers share information with fellow customers frequently before making purchase decisions (Gong, 2018). This allows researchers to identify consumers’ community engagement behaviors clearly. This paper uses one of the largest social platforms in China, Baidu Tieba, on which many smartphone brands (Xiaomi, Meizu, Huawei, Apple, OPPO, VIVO, and LeTV) have their dedicated communities (Liao et al., 2021a). Baidu users can participate in multiple brand communities simultaneously, making it easier to observe the consumers’ MBCE. We observe the consumers’ MBCE through their engagement (mainly posting and reposting behaviors in various brand communities). Posting and commenting behaviors of 10,000 users are obtained with the help of a self-designed Python crawler. Among them, we determine that 2,138 users have posting or commenting behaviors in two or more competing smartphone brand communities. Around 21.38% users presented MBCE behaviors. We need to analyze the posts and comments to uncover the manifestation and types of MBCE. Because these users produce 10,457 posts and 57,668 comments and it is much infeasible to analyze all the data, we decided to choose 200 random users with 1,374 posts and 4,832 comments for formal analysis. Note that which community each post or comment comes from are recorded to identify the users’ MBCE. We simultaneously used participating observation, non-participating observation, and email/instant messaging interviews to improve the accuracy and representativeness of data during the implementation of netnography (Xun and Reynolds, 2010).

### Analysis and findings

To understand the meaning of interaction within a community better, netnography requires the researcher to be knowledgeable about online communities. The authors particularly focused on the smartphone brand community in Baidu Tieba and joined several brand community members’ QQ groups to communicate with them directly to understand their behaviors and motivations. Based on the users’ activities in brand communities, the authors summarized the types of MBCEs into three categories: (1) information-oriented MBCE, (2) social-oriented MBCE, and (3) oppositional MBCE, thus analyzing the typical consumer characteristics among them.

#### Information-oriented multi-competing brand community engagement

After observing and sorting out users’ engagement behavior in multiple smartphone brand communities, we determine that many consumers’ engagements in multiple competing brand communities are driven by the need to search for information. They are likely to seek information in various brand communities while they are facing a purchase decision. Some consumers typically participate in multiple smartphone communities to express their views or ask for recommendations in one of the smartphone communities, examples of which are illustrated in Table 1.

**Table 1:** Multi-brand community engagement in a purchase decision context.

User	Posting content and the related community	Related community
ne****	【追求源于热爱】魅族Pro6s和Pro6 Plus选哪个??? 本人不玩大型3d游戏, 但经常逛论坛, 看贴吧, 偶尔看看美剧, 日剧, 300元的差距, soc., 2k屏幕, 都用过的魅友给点建议【魅族】 [Pursuit from love] which phone should I choose, Meizu Pro6s or Pro6 Plus?? I do not play large 3d games, but often visit forums, read postings, and occasionally watch American and Japanese TV series. Given the 300 yuan price difference between them, soc., 2 k screen, hoping friends who used both of them to give some advice [Meizu community] ***Note: All consumer postings are excerpts of the original text*, *typos or grammatical errors are still in accordance with their origin (same below)***	Xiaomi, Meizu
咖****	#问答#3000元左右旗舰机选哪个 本来想给爸买个红米Note4x的不过想想还是给老爸买旗舰机吧, 一加3t,小米Note2, 华为荣耀v9, 魅族Pro6都选6加128版本, 外观, 实际使用流畅度(软件开启速度, 软件运营流畅度, 开机速度, 指纹解锁速度), 拍照, 音质, 通话质量, 系统, 屏幕质量哪个好 【小米】 #Q & A# Which flagship phone around 3,000 yuan to choose I wanted to buy a Redmi Note4x for my dad, but I finally decided to buy a flagship phone for him. Considering the 6 + 128 version, Oneplus 3 t, Xiaomi Note2, Huawei Glory v9, and Meizu Pro6, when it comes to appearance, actual use smoothness (software open speed, software operation smoothness, boot speed, fingerprint unlock speed), photo quality, sound quality, call quality, and system, which phone is better? [Xiaomi community]	Xiaomi, Huawei Glory
Cry****	最近想入一款1,000 + 的手机, 看了魅族, 荣耀, 还是最心水小米, 还不知道那款好。求推荐性能和外观都比较好的小米。【小米】 Recently I plan to buy a phone with a budget of about 1,000 yuan. I am searching for information about Meizu, Glory, and especially Xiaomi, but still do not decide which one to choose. I’m looking for recommendations to choose phones among Xiaomi in terms of performance and appearance [Xiaomi community]	Xiaomi, Huawei Glory and Meizu

**Figure 1:**
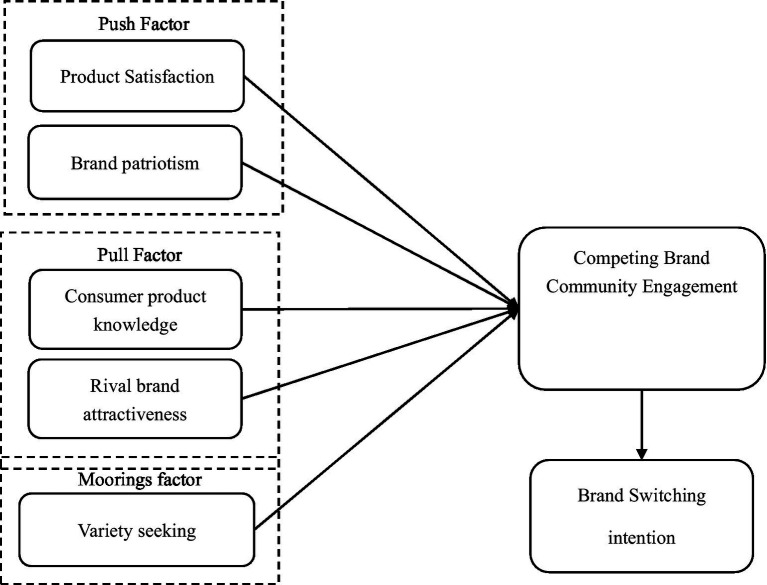
Research framework.

User “ne****” revealed that s/he participated in several brand communities just to obtain information about various products and make a purchase decision. These information-oriented users already have a clear buying need and participate in different brand communities and compare different brands or products. This type of user has not yet established brand preferences and is a potential customer that companies can compete with each other to acquire.

There is a group of consumers who are dissatisfied with their previous brands and are willing to switch brands. They start to pay attention to other smartphone brands and join the rival brand community. This is a type of competing brand community engagement for brand switching. Typical examples are illustrated in Table 2:

**Table 2:** Multi-brand community engagement in a brand-switching context.

User	Posting content and the related community	Related community
怪****	脱坑了, 用了将近两年的mx4, 转战zuk【ZUK】	Meizu, ZUK
	I gave up the mx4 which was used for nearly 2 years and planned to try Zuk [ZUK community]	
释****	#已从米粉转米黑#呵呵, 连王者荣耀都带不动, 老子还不如买oppor9, 不会再买小米手机【小米】	Xiaomi, OPPO
	# I have turned from fans of Xiaomi to anti-fans # Huh, Xiaomi phone even cannot operate the king of glory fluently, I might as well buy Oppo r9 and will not buy Xiaomi phone [Xiaomi community]	
陨****	我这M4用了快两年了, 期间屏碎了一次花了540(心疼我第一次的奖学金), 也有过屏幕突然自己乱点的情况, 自己打开各种软件, 差点给别人转了钱, 现在想想也是心惊肉跳, 现在手机满目疮痍了, 后盖也换过一次了, 想说说这两年的感受, 小米确实是一款不错的手机, 不过还有诸多方面需要改善, 耗电太快, 手机过烫, 且经常更新, 说实话有些更新挺不错的, 但是有些可有可无的更新真的没必要, 倒不如把那些更新放到一块更新一次, 以上纯属个人感觉, 不对之处还请各位老铁勿喷另外vivo没用过, 不知道咋样【小米】	Xiaomi, VIVO
	I have been using M4 for almost 2 years, during which the screen shattered and I spent 540 yuan (my first scholarship) to fix it. Its screen failed suddenly, a variety of software was opened automatically, and then almost transferred the money to others. Thinking about it also makes me heartbreaking. Now the phone is in poor condition, and the back cover has also been replaced once. I want to talk about the feelings of Xiaomi these two years: Xiaomi is indeed a good phone, but many aspects should be improved, such as the rapid power consumption, high-temperature issue, and too frequent update. To be honest, some updates are unnecessary. These comments are just personal feelings. I do not use Vivo and do not know the details about it [Xiaomi community]	

“怪****” expresses his/her disengagement with the Meizu brand and decides to join the ZUK community. This post was published in the ZUK community, possibly to gain acceptance from ZUK users because new members face legitimacy challenges when entering a new community (Thompson et al., 2016). “释****” expresses his/her disappointment with Xiaomi and willingness of switching to other communities directly in the Xiaomi community. “陨****” also expresses the same willingness to switch, but in a moderate tone compared with the previous user. These users are disappointed with their focal brands, then express their willingness to brand switching.

#### Social-oriented multi-competing brand community engagement

We observe a category of users who do not show their favor of a specific brand but participate in different brand communities to answer users’ questions about brands and products, showing their knowledge and understanding of the entire category. They pursue influence and status in the community to satisfy self-expression. Therefore, we define this type of engagement behavior as social-oriented MBCE.

The essential characteristic of this group of consumers is that they define themselves as category fans and do not care about competing relationships between brands. According to Heider’s cognitive equilibrium theory, if consumers perceive two brands in competition, it is difficult for them to form a coherent perception (Heider, 1946). However, specific brands are viewed as just one part of the smartphone brands for participants in socially oriented multi-competing brand communities and they have complementary relationships with each other, showing their own strengths. Typical examples are as follows:

These two users from multiple smartphone communities participated in discussions in various smartphone communities, showing their knowledge and familiarity with the smartphone brands. For example, comments of “爱****” on Honor 8 and the Lumia 1,520 in different communities show that he has knowledge of multiple smartphones and can provide professional answers.

#### Oppositional multi-competing brand community engagement

There is a group of consumers who are extremely fond of a particular brand but participate in other brand communities to “show off” their focal brand and disparage rival brands or products. The rival brand community engagement means that the user has a deep emotional attachment to the focal brand and participates in other competing communities to promote the focal brand or disparage rival brands. We refer to this type of multi-competing brand engagement as oppositional MBCE. Their rival brand community engagement aims to defend the focal brand and criticize rival brands.

Consistent with the observation by Muniz and O’guinn (2001), users tend not to only be loyal to the focal brand community, but also usually carry oppositional loyalty to rival brands. Sociological research has also shown that competition between groups reinforces the social identity of people within their groups, after which they may adopt more hostile attitudes toward the rival group (Cikara et al., 2011). The study has also indicated that competition between groups reinforces the social identity of people within their respective groups, then they adopt more hostile attitudes toward the rival group. Similar research also indicates that intergroup competition leads group members to prefer their own group’s products (Rabbie and Wilkens, 1971). The collective pride or group centrism that arises within a brand community is generally a source of group conflict. Collective pride often stems from comparisons with other groups and this type of user disparages and mocks rival groups to demonstrate its superiority further (Ewing et al., 2013).

The authors also observed that such consumers are especially eager to participate in important events such as new product launches in other brand communities, as well as engage in posts in rival communities where the focal brand is used as a comparison. They enjoy deriding, mocking, and teasing other’s new products at these events. Typical examples are as follows:

These users engage in rival communities to make hostile comments, which is a type of oppositional behavior. Their reason for oppositional MBCE is to show their support of the focal brand out of the emotional attachment to this focal brand.

## Study 2: A survey study on multi-competing brand community engagement

Study 1 initially explored the types and motivations of consumers’ MBCE from a qualitative perspective. The potential impact of these three types of multi-competing community engagement is influenced by the consumers’ perceptions of the focal brand, rival brands, product categories, and possibly consumers’ own factors. Study 2 aims to test the aforementioned motivations by conducting a survey.

### Hypothesis development

Multi-competing brand community engagement is probably similar to brand switching. Moon (1995) first proposed the push-pull-mooring (PPM) model in migration and Bansal (2005) determined that migration is extremely similar to brand switching, applying this theoretical framework to explain consumers’ brand switching. The “push” factors include low satisfaction, low value, and high price perceptions. The “pull” factors arise mainly from rival brand attractiveness. These two factors directly affect the consumers’ willingness to switch and purchase behavior. The “mooring” factors include switching costs and variety seeking, which moderate the influence of these two types of factors. Consumers’ decision of changing a smartphone brand usually involves internal, external, and environmental factors, the PPM model provides a good theoretical lens for an in-depth understanding of the drivers of smartphone brand switching (Liao et al., 2021b). The PPM model also provides a proper model to study the generation of MBCE.

This study identifies several important factors combined with the preliminary findings of Study 1. First, Study 1 indicates that product satisfaction and brand patriotism may be important “push” factors influencing engagement in multi-brand communities, as Study 1 indicates that some consumers participate in other communities because they are dissatisfied with a brand after using it and want to find alternative brands. Some consumers may participate in other competing communities because of their love for their own brand. The concept of brand patriotism was proposed based on patriotism in political psychology, which indicate consumers’ sense of identity and attachment to the brand. They found that brand patriotism is an important variable to predict focal brand consumers’ attitudes toward the outside brand community. As the community is a group concept, it is worth investigating whether this concept can be applied to community contexts (Bellezza and Keinan, 2014). Coupled with Study 1, the attractiveness of other competing brands and the consumers’ product knowledge may be an important “pull” and variety seeking is a “mooring” factor for consumers’ engagement in multiple brand communities. We also explore whether MBCE leads to higher brand-switching intention.

Brand satisfaction and brand patriotism are negatively related to brand-switching willingness (Bansal, 2005; Bellezza and Keinan, 2014). Therefore, both of them may negatively influence engagement in competing brand communities. Drawing from Study 1, consumers participate in competing brand communities because they want to oppose the rival brand, and we refer to this type of engagement as oppositional “MBCE.” Product satisfaction and brand patriotism may have various effects on MBCE for the reason that product satisfaction and brand patriotism do not have the same affective level for the brand. Satisfaction is a rational judgment from consumers that involves consumers’ comparison between the actual performance of this product and their expectation, but it does not affect the user’s judgment of other brands. Therefore may not affect the willingness of the oppositional MBCE (Wang et al., 2018). Nevertheless, brand patriotism is a positive consumer sentiment toward the brand, which involves self-brand identification and connection (Saxena and Dhar, 2021). Drawing from social identity theory, consumers usually have preferences for in-groups but stereotypes or even negative perceptions of out-groups. As a result, brand patriotism may lead to an oppositional MBCE. This research proposes the following hypotheses:

*H1*: *Product satisfaction negatively relates to the possibility of rival brand community engagement*.

*H2*: *Brand patriotism negatively relates to the possibility of rival brand community engagement*.

The rival brand attractiveness may motivate consumers to participate in other brand communities to learn about the brand, providing the possibility for consumers to supplement their product knowledge (Thompson et al., 2016). Thus, the attractiveness of other products may lead consumers to participate in competing brand communities. When consumers do not have enough product knowledge, they may also participate in other competing brand communities to supplement their product knowledge, which is the so-called “pull” factor. Therefore, the following hypothesis are proposed:

*H3*: *Rival brand attractiveness positively relates to the possibility of rival brand community engagement*.

*H4*: *Consumer product knowledge negatively relates to the possibility of rival brand community engagement*.

The consumers’ characteristics may play a “mooring” role. Prior studies have indicated that self-presentation gratifications affect users’ platform-swinging behaviors, which refer to their routine use of multiple social media platforms (Tandoc et al., 2019). We argue that the consumers’ propensity for seeking variety may promote consumer engagement in multiple competing brand communities, therefore giving rise to the following hypothesis:

*H5*: *The propensity for variety seeking positively relates to the possibility of rival brand community engagement*.

Higher participation in a product category community leads to lower switching costs and increases the possibility of adopting new products (Thompson et al., 2019). It is because participation in brand communities of the same product category exerts social pressure on the focal members, motivating them to keep up with members of various communities and thus mitigating the impact of switching costs. Participation in multi-competing brand communities also allows consumers to learn about the advantages of products from different brands, which may enhance their willingness to brand switching. Thus, the following hypothesis is proposed:

*H6*: *The number of multi-competing brand communities that consumers participate in is positively related to the consumers' brand-switching willingness*.

### Methodology

#### Data collection

Consistent with Study 1, the authors invite users of smartphone brand communities in Baidu Tieba to participate in a survey. A total of 457 questionnaires were received, 31 invalid questionnaires were deleted, and 426 valid questionnaires were obtained. In this questionnaire design, we listed nearly 10 major smartpho are currently available on the market and ask respondents which smartphone brands they currently use (those consumers who use two or more different smartphone brands at the same time are finally excluded). Second, we also listed the aforementioned smartphone brands and asked them whether they participated in the online communities (including Baidu Tiebaing and other types of online communities) of these smartphone brands in the last 3 months. Next, we used a seven-point Likert scale to measure the latent constructs. Finally, respondents are asked for demographic information.

The authors define the smartphone brand they were using as the focal brand and other brands as rival brands to identify the number of users who participate in rival smartphone brand communities. Results show that the number of people who do not participate in the rival brand community is 305 (71.6%), while the number of people who participate in the rival brand community is 121 (28.4%), indicating that MBCE is a relatively common phenomenon. The number of those who participate in one other rival brand community is 63, the number of those who participate in two rival brand communities is 35, while the number of those who participate in three or more rival brand communities is 23. The valid samples comprise 279 (65.5%) males and 147 (34.5%) females; 98 (23%) are under 23 years old, 227 (53.3%) are 24–30 years old, and 101 (23.7%) are 31 years of age or older.

### Variable measurement and reliability testing

The measures of “product satisfaction,” “attractiveness of rival brand,” “product knowledge,” “variety seeking,” and “brand switching willingness” are from Bansal (2005). The measures of “brand patriotism” are adopted from Bellezza and Keinan (2014). One item exists for brand switching intention: “I will probably choose another brand the next time I buy a smartphone.” The measurement items are shown in Table 2.

For the reliability test, the authors examined the internal consistency reliability and the combination reliability. Table 2 shows that the Cronbach’s α values for product satisfaction, brand patriotism, rival product attractiveness, product knowledge, and variety seeking are 0.816, 0.937, 0.912, 0.884, and 0.876, respectively. All values are above or close to around 0.8, indicating that the internal consistency of each construct is high. All the constructs show well combined reliability (all values of combined reliability exceeded 0.88). Then, we also examine the convergent validity and discriminant validity. First, using validated factor analysis to test convergent validity. Results show that (1) the factor loadings of the question items are all greater than 0.800; (2) Overall model fit indices (χ2 (134) = 283.466, χ2/df = 2.115, *p* < 0.001, RMSEA = 0.064, CFI = 0.958, NFI = 0.954, IFI = 0.947, GFI = 0.923) are satisfactory. The measurement has good convergent validity.

Table 3 reports the correlation coefficients between the variables. Discriminant validity is mainly judged by comparing the mean extracted variance of each construct with the square of the correlation coefficient between the other variables. If the average extracted variance (AVE) of a variable is higher than the square of the correlation coefficient between the other variables, all measures possessed adequate judgmental validity. By comparing the magnitude of the average extracted variance in Table 2 and the squared correlation coefficients of the variables in Table 3, the measurement in this paper has good discriminant validity. Next, model estimation is performed to test the hypotheses.

**Table 3:** Social-oriented MBCE.

User	Posting content and the related community	Related community
爱****	某用户:在华为荣耀8与5s间纠结, 求解答, 谢谢了	Xiaomi, Nokia, Meizu
	爱问小盗:我觉得荣耀八可能漂亮点, 配置小米好点, 拍照也应该是小米这台好, 我买过荣耀八用了两天退了买了小米, 感觉良好 【小米】	
	A user: I cannot decide to choose Huawei Honor 8 or 5 s, so come here to ask for some advice, thank you	
	爱问小盗: I think the Glory 8 looks better than Xiaomi, but the configuration and photographic function of Xiaomi is better than the Glory 8. I bought Glory 8 and used it for 2 days. Then I returned it to buy Xiaomi, and now I feel good [Xiaomi community]	
爱****	某用户:『02–03|提问』1,520拍照在现在处于什么水平, 和国产的比呢	Xiaomi, Nokia, Meizu
	爱问小盗:不会手动像素一般般, 会手动还是高端【Lumia吧】	
	A user: “02–03|Questions” What about the photographic level of model 1,520 compared with a domestic phone?	
	爱问小盗: If you are bad at taking photos, pixel level is ordinary. If you do well in taking photos, the quality of your photos will be good [Lumia community]	
笨****	某用户:羡慕嫉妒恨。开启284个APP, 毫无卡顿, 你们MIUI做得到吗	Meizu, Xiaomi, Microsoft, Google
	笨鸟多只:老子pixelxl照样, 另外那不是后台, 那只是后台界面, 撒比楼主【小米吧】	
	A user: jealousy and envy. Open 284 APPs, no lag, can MIUI do it?	
	笨鸟多只: My Pixel xl can do it as well, in addition, that is not the backstage but the backstage interface [Xiaomi community]	
笨****	某用户:750收了个Nexus 6不知道算不算亏	Meizu, Xiaomi, Microsoft, Google
	笨鸟多只:不亏【Nexus吧】	
	A user: I spent 750 yuan buying a Nexus 6 and do not know if it is a loss	
	笨鸟多只: Not a loss [Nexus community]	

### Hypothesis testing

First, we explore factors that influence the possibility of the consumers’ competing brand community engagement. We coded the dependent variable as a dummy variable, where 1 represents engagement in competing brand communities and 0 represents no such engagement. Since the dependent variable is a dichotomous variable, Model 1 uses logistic regression to test the hypotheses about the possibility of competing brand community engagement. Results are shown in Table 4.

**Table 4:** Oppositional MBCE.

User	Posting content and the related community	Related community
神****	魅族 完了 等倒闭吧【魅族】	Meizu
	Meizu is down and it is waiting for its closing down.[Meizu community]	
石****	如果雷布斯拿魅族当追逐对手的话早已经倒闭好几遍了【魅族】	Meizu
	If the leaders regard Meizu as a rival, it would have already closed down several times [Meizu community]	
逆****	进魅族吧逛一圈顿时感觉我大小米简直意气风发【魅族】	Xiaomi, Meizu
	Browsing the Meizu bar suddenly makes me feel my Xiaomi is simply nice.[Meizu community]	
ll****	我年前闲鱼550入手zuk1用到现在, 吊打魅4, 电池顶用4妹两个还多, 玩农药就不知道啥叫卡顿【魅族】	Meizu, ZUK
	I paid 550 yuan to buy Zuk1 years ago from Xianyu and have used it until now, hanging Meizu 4. The battery of Zuk is twice better than Meizu4 and it can operate King of glory with no lag [Meizu community]	
白****	#小米5c#垃圾小米 谁买谁傻	Xiaomi, Huawei
	没一样拿手的技术,只会耍猴。 切记各位必要被米水军忽悠。小作坊毫无技术水平,七拼八凑的垃圾,骗钱	
	# Xiaomi 5c # Xiaomi is junk and only idiots will buy it. There is no one technique that Xiaomi is good at and it only cheats on customers. Remember that do not be fooled by the paid Internet trolls. The small workshop has no technology, just like garbage, cheating money	

Results indicate that (1) the rival brand attractiveness has a positive effect on competing brand community engagement, which indicates that the rival brand attractiveness is an important motivation forcing consumers to participate in competing brand communities. (2) The more product knowledge consumers have, the more likely consumers will participate in a multi-competing brand community (Tables 5–8). The result is contrary to our hypotheses for the following reasons: First, consumers possibly acquired product knowledge by participating in multi-competing brand communities. Second, as Study 1 indicated, some consumers prefer answering users’ questions in rival communities precisely because of their abundant product knowledge, wherein they can gain social influence in the communities and enhance their community status. (3) This study is consistent with previous research from Garga et al. (2019), which indicates the attractiveness of the opportunity to inspect and expand the number of alternatives is dependent in part on the consumer’s ability to sort efficiency through information search. (4) Product satisfaction has no significant effect on the consumers’ competing brand community engagement. (5) Variety seeking does not have a significant effect on the consumers’ competing brand community engagement. This claims that competing brand community engagement is not motivated by the propensity for variety seeking. (6) Results indicate that brand patriotism negatively influences the consumers’ competing brand community engagement. Coupled with the differences in the effects of satisfaction and brand patriotism, we can draw an interesting conclusion that deep brand emotions (e.g., “brand patriotism”) are more likely to lead to brand community loyalty. Consumer satisfaction is based on the practical product effect compared with personal expectations, which is relatively rational and does not lead to brand community loyalty. When satisfied consumers are attracted to rival brands, they may leave the focal brand community. Finally, we tested the effect of the number of consumers’ competing brand community engagement on brand switching intention. The linear regression indicates that it has a coefficient of 0.136*, t = 2.079. This demonstrates that the width of MBCE leads to enhanced consumer brand intention.

**Table 5:** Types and Characteristics of MBCE.

Types of MBCE	Typical people	Typical behavioral characteristics
Information-oriented MBCE	New market entrants, people dissatisfied with the original brand	Ask questions and seek information; often appear to compare different brands and products
Social-oriented MBCE	Category enthusiast, expert	They are not loyal to a particular brand but are deeply involved in multi- communities, they are ready to answer product inquiries from members in different communities and give relatively objective comments on the comparison of each brand, and consider themselves highly knowledgeable about each brand
Oppositional MBCE	Brand loyalists of a brand, community maintainers of a brand	Promote the preferred brand, deride and mock the competitor brands

**Table 6:** Results of validation factor analysis.

Construct	Item	Factor load
Product Satisfaction	I am satisfied with the phone I am currently using	0.867
Cronbach’s alpha =0.816		
CR = 0.914	My current phone meets my expectations	0.918
AVE = 0.781	I think I have a good phone for this	0.865
Brand Patriotism	I love my smartphone brand	0.877
Cronbach’s alpha = 0.937	I am proud to use my phone brand	0.923
CR = 0.949	I have an attachment to my phone brand	0.884
AVE = 0.789	I am proud to be a member of my smartphone brand users	0.903
	Seeing the brand logo (LOGO) of my smartphone brand makes me feel close	0.853
Attractiveness of Rival brands	I think other phone brands are also attractive to me	0.834
Cronbach’s alpha = 0.912		
CR = 0.897	I would be happier if I could buy or use another phone brand	0.911
AVE = 0.745	I think it’s better to buy other phone brands than my current phone brand	0.842
Product Knowledge	I have extensive knowledge of smartphones	0.945
Cronbach’s alpha = 0.884		
CR = 0.928		
AVE = 0.867	I understand all aspects of smartphones	0.917
Variety Seeking	I like to use a brand for a long time rather than switching to another brand I am not familiar with (reverse)	0.881
Cronbach’s alpha = 0.876		
CR = 0.901	If I like a brand, I rarely buy another brand because I want to try something new (in reverse)	0.874
AVE = 0.752	I am very careful when trying new, different brands (in reverse)	0.846

**Table 7:** Variable correlation matrix.

Variables	1	2	3	4	5	6	7
1. Product satisfaction	1						
2. Brand patriotism	0.438^**^	1					
3. Rival brand attractiveness	0.149	0.199^**^	1				
4. Product knowledge	0.511^**^	0.556^**^	0.181^*^	1			
5. Variety seeking	0.489^***^	0.592^**^	0.290^**^	0.593^**^	1		
6. Number of multi-competing brand community engagement	0.012	−0.067	0.220^**^	0.1	0.008	1	
7. Brand switch	−0.342^**^	−0.145^***^	0.239^**^	0.092^**^	0.084^***^	0.105^***^	1
8. Mean	4.961	4.783	4.431	4.271	4.587	0.474	3.611
9. Standard deviation	1.251	1.246	1.453	1.428	1.348	0.86	1.413

*p < 0.05, **p < 0.01, ***p < 0.001.

**Table 8:** Factors influencing community engagement of competing brands.

Variables	MBCE
	Model 1
1. Product satisfaction	−0.023
2. Brand patriotism	−0.181***
3. Attractiveness of rival brands	0.347**
4. Product knowledge	0.054*
5. Variety seeking	−0.228
6. Gender (Male = 1, Female = 0)	0.103*
7. Age	0.391
8. Income	0.246
Cox&Snell R square	0.257
Nagelkerke R square	0.264
−2Loglikelihood	3467.365

*p < 0.05, **p < 0.01, ***p < 0.001.

## Discussion

The first study identifies three types of MBCE behaviors through netnography: information-oriented; social-oriented and oppositional MBCE. A study was then conducted to reveal the motivations for MBCE through questionnaire research based on the classical “push-pull-mooring” model. Our results indicate that (1) consumers may participate in other competing brand communities for the reason that they are attracted by other competing brands. (2) The higher product knowledge consumers have, the more likely they are to participate in competing brand communities. This may indicate that opinion leaders or expert consumers are more inclined to participate in multi-competing brand communities and they have lower brand loyalty. (3) The number of competing brand community engagements is positively related to the willingness to switch brands. We determine that product satisfaction does not necessarily make consumers maintain loyalty to the brand community, while brand patriotism is an important factor in forming loyalty to the brand community and oppositional loyalty to the rival brand community.

### Theoretical contribution

First, this study expands the research on the consumers’ brand community engagement. Previous literature has conducted relatively adequate research on the motivation and impact of consumer engagement in a single brand community engagement (Kumar and Kumar, 2020; Yuan et al., 2020). However, social media platforms enable consumers to participate in multiple virtual brand communities simultaneously (Phua et al., 2017; Pelletier et al., 2020). For example, Liao et al. (2022) examined the effect of four values on the consumers’ community-swinging behaviors. However, consumer engagement in multi-competing brand communities has not been adequately emphasized and studied (Thompson et al., 2016, 2019). This study takes a holistic view of the consumers’ community engagement behaviors and considers the influence of rival brand communities on community members, which provides a more comprehensive understanding of consumer community engagement behavior in academia and enriches brand community engagement literature (Tan and Lee, 2015).

Second, as one of the pioneer efforts to break through the previous perspective concentrating on the internal brand community, this study reveals the influence of external competing brand communities on the focal brand community. Although studies on brand communities have yielded relatively rich results, previous brand community studies have often focused on the internal aspects of a community, rarely considering the influence of external competing brand communities on this brand community (Liao et al., 2022). The trans-competing brand community engagement behaviors among members bring intrinsic dynamics to the evolution of interactions among brand communities (Thompson et al., 2016). This study fills a key research gap by exploring the influence of the external environment on brand communities, especially the influence brought by other competing brand communities from the perspective of MBCE. Thus, our study breaks through the internal perspective and develops a combined internal and external perspective on brand communities.

Finally, this study distinguishes the types and motivations of consumer engagement in competing brand communities. Previous research has mentioned the consumers’ behaviors of participating in multiple competing brand communities simultaneously and this study further broadens the understanding of such behaviors (Thompson et al., 2016, 2019). For information-oriented multi-competing brand community participants, the brand may be incompatible with such consumers, and they are searching for a final choice of a satisfactory brand or product (Wang et al., 2019). However, for social-oriented multi-competing brand community participants, brands are not as important to them (Kumar and Kumar, 2020). On the contrary, they have complete knowledge of the category and can easily move between brands and try different brands because they deemed it as not an incompatible relationship between brands. Finally, for oppositional MBCE, customers may dislike rival products (including the meaning embedded in the products) and firmly defend their own group because of love for the focal brand (Kuo and Hou, 2017; Liao et al., 2021a). It is not only an incompatible choice of product, but also an incompatible choice of group identity for them. Thus, the research determines that consumers have multi-level preferences for competing brand community engagement, providing a foray into better understanding the behavior of groups and users within communities.

### Managerial implication

This paper provides several important practical implications for companies to manage their brand communities. First, brand managers should manage brand communities in terms of customer inflow and churn. Our research shows the dynamic nature of community membership. Members of this community would possibly transfer to other communities in the future to become consumers of rival brands and vice versa. Therefore, companies should closely monitor the community engagement of consumers and identify those who may switch to other communities. For example, if consumers suddenly or gradually stop participating in the focal community and become active in other communities, companies should promptly analyze the reason and use corresponding methods to pull them back (Bowden et al., 2018; Haverila et al., 2020; Kumar, 2021). Some users of rival brands may also try to participate in the community of the focal brand (Thompson et al., 2016). These consumers may have the potential need to switch brands, and companies should target to help these consumers recognize the brand and provide them with the information they need, wherein companies can convert consumers of rival brands into their own consumers.

Second, given that differences in consumers’ needs for products and brands exist, brand managers should consider the cost and value of maintaining different kinds of users and attempt to apply category management (Fernandes and Moreira, 2019). Some users in the community may be product enthusiasts who are not loyal to brands and others are brand enthusiasts with a high level of commitment to the brand (Rauschnabel et al., 2015; Hook et al., 2018). For product enthusiasts, the brand may not be a very influential factor while the product itself is important. Instead of being loyal to a single brand, product enthusiasts tend to wander among multiple brands and treat different smartphone brands more rationally and fairly (Dodd et al., 1996). For one brand, it costs more to facilitate product enthusiasts’ brand loyalty. Brand enthusiasts are unwilling to try different brands, but they define themselves as consumers of a certain brand with a stronger brand identity and group identity (Dodd et al., 1996; Kwon and Mattila, 2015). When they believe that the group of rival smartphone brands is denigrating or overly boastful about the focal brand, they will take the initiative to boycott the brand and criticize it in the rival brand community (Kuo and Hou, 2017; Liao et al., 2021a). Such consumers are the defenders of the brand. Therefore, brands should foster OMBCE, where such consumers may help the brand toward maintaining its reputation and also attract members from other communities.

### Future research

This paper is a first step that explored the motivations and types of consumer MBCE and it contributes to a nuanced understanding of competing brand community engagement. Future exploration can be conducted in the following ways to advance this research stream. First, the impact of various types of competing brand community engagement is valuable. For example, it is interesting to explore the users’ reactions when their brand community is invaded by consumers of rival brands communicating the benefits and advantages of rival brands. Second, previous studies have revealed the heterogeneity of brand community members, which means that they may participate in brand communities to satisfy different needs (Ozboluk and Dursun, 2017; Liao et al., 2019). Some of these users are brand loyalists, some participate in brand communities just to learn about the brand, and some are even brand opponents, which challenges the traditional notion that all users in brand communities are brand loyalists. Consequently, it is worthy for scholars to explore firm strategies that effectively manage heterogeneous user groups in a brand community.

## Data availability statement

The raw data supporting the conclusions of this article will be made available by the authors, without undue reservation.

## Ethics statement

Written informed consent was obtained from the individual(s) for the publication of any identifiable images or data included in this article.

## Author contributions

KH, JL, FL, and HS contributed to writing, reviewing, and editing the manuscript. JL proposed the framework and performed data analysis. All authors contributed to the article and approved the submitted version.

## Funding

This project was supported by National Natural Science Foundation of China (NSFC) (Nos. 72272061 and 71802097), Ministry of Education of Humanities and Social Science Project (No. 22YJC630077), Philosophy and Social Sciences Planning Program of Guangzhou (Nos. 2021GZYB05 and 2022JDGJ06), Research Institute on Brand Innovation and Development of Guangzhou (No. 2021CS05), Jinan University Management School Funding Program (No. GY21013), and Institute for Enterprise Development, Jinan University, Guangdong Province (Nos. 2021MYZD04 and 2020CP03).

## Conflict of interest

The authors declare that the research was conducted in the absence of any commercial or financial relationships that could be construed as a potential conflict of interest.

## Publisher’s note

All claims expressed in this article are solely those of the authors and do not necessarily represent those of their affiliated organizations, or those of the publisher, the editors and the reviewers. Any product that may be evaluated in this article, or claim that may be made by its manufacturer, is not guaranteed or endorsed by the publisher.
